# Epithelial neoplasia coincides with exacerbated injury and fibrotic response in the lungs of *Gprc5a*-knockout mice following silica exposure

**DOI:** 10.18632/oncotarget.5532

**Published:** 2015-10-02

**Authors:** Xiaofei Wang, Dongliang Xu, Yueling Liao, Shuangshuang Zhong, Hongyong Song, Beibei Sun, Binhua P. Zhou, Jiong Deng, Baohui Han

**Affiliations:** ^1^ Department of Pulmonary Medicine, Shanghai Chest Hospital, Shanghai Jiao Tong University, Shanghai, China; ^2^ Department of Pulmonary Medicine, Ruijin Hospital, School of Medicine, Shanghai Jiao Tong University, Shanghai, China; ^3^ Key Laboratory of Cell Differentiation and Apoptosis of Chinese Minister of Education, Department of Pathophysiology, Shanghai, China; ^4^ Shanghai Key Laboratory for Tumor Microenvironment and Inflammation, Shanghai Jiao Tong University School of Medicine, Shanghai, China; ^5^ Translation Medicine Center, Shanghai Chest Hospital, Shanghai Jiao Tong University, Shanghai, China; ^6^ Department of Molecular and Cellular Biochemistry, Markey Cancer Center, University of Kentucky College of Medicine, Lexington, KY, USA

**Keywords:** SiO_2_, Gprc5a, fibrosis, lung cancer, neoplasia

## Abstract

Exposure to crystalline silica is suggested to increase the risk for a variety of lung diseases, including fibrosis and lung cancer. However, epidemiological evidences for the exposure-risk relationship are ambiguous and conflicting, and experimental study from a reliable animal model to explore the relationship is lacking. We reasoned that a mouse model that is sensitive to both lung injury and tumorigenesis would be appropriate to evaluate the exposure-risk relationship. Previously, we showed that, *Gprc5a*^−/−^ mice are susceptible to both lung tumorigenesis and endotoxin-induced acute lung injury. In this study, we investigated the biological consequences in *Gprc5a*^−/−^ mouse model following silica exposure. Intra-tracheal administration of fine silica particles in *Gprc5a*^−/−^ mice resulted in more severe lung injury and pulmonary inflammation than in wild-type mice. Moreover, an enhanced fibrogenic response, including EMT-like characteristics, was induced in the lungs of *Gprc5a*^−/−^ mice compared to those from wild-type ones. Importantly, increased hyperplasia or neoplasia coincided with silica-induced tissue injury and fibrogenic response in lungs from *Gprc5a*^−/−^ mice. Consistently, expression of MMP9, TGFβ1 and EGFR was significantly increased in lungs from silica-treated *Gprc5a*^−/−^ mice compared to those untreated or wild-type ones. These results suggest that, the process of tissue repair coincides with tissue damages; whereas persistent tissue damages leads to abnormal repair or neoplasia. Thus, silica-induced pulmonary inflammation and injury contribute to increased neoplasia development in lungs from *Gprc5a*^−/−^ mouse model.

## INTRODUCTION

Chronic respiratory exposure to crystalline silica (CS), either occupational or environmental, causes an accumulation of injuries with activation of inflammatory cells in the lung, and leads to tissue damage. Persistent tissue damage and abnormal repair ultimately leads to fibrosis and a variety of chronic pulmonary diseases such as silicosis [1]. An estimated 200,000 miners and 1.7 million non-mining workers have occupational exposures to inhaled silica in the United States alone [2]. Data from high-quality epidemiologic studies in Asia, Europe, and North America consistently show positive associations between lung cancer and particulate matter (PM) exposure or other forms of air pollution, and these correlations persist after adjustment for important lung cancer risk factors, such as tobacco smoking [3]. Particles of silica are an important component of PM 2.5, a designation for fine particulates in the air, especially in the western regions of mainland China [4].

Fine-grained quartz and silica are known to cause silicosis in humans, and the co-occurrence of lung cancer and silicosis implies a causal relationship between silicosis and lung cancer. However, other studies show no evidence of an exposure-response relationship for silica dust and the severity of silicosis, lung cancer or death [5, 6]; and there is lack of experimental study from mouse or hamster models to show an elevated lung cancer risk in response to CS exposure. For the development of silicosis, extrinsic factors such as duration, total exposure, and content of free crystalline silica are critical determinants [2]. However, intrinsic (genetic) factors also influence the susceptibility to silicosis. For example, the human leukocyte antigen (HLA) haplotype has been associated with silicosis [7] and pneumoconiosis [8]. In addition, gene polymorphisms of pro-inflammatory cytokines (e.g. TNFα, IL-1β) have been associated with silicosis [9–11]. Thus, both extrinsic and intrinsic factors are important in lung cancer development. We reasoned that the causal relationship of silica exposure to lung neoplasia might be reproduced in a mouse model that is sensitive to both silica-induced fibrosis and lung tumorigenesis.

G-protein coupled receptor family C group 5 type A (*GPRC5A*), also known as *RAIG1* or *RAI3*, is a retinoic acid-inducible gene that is predominately expressed in type I and type II epithelial cells of the lung [12–15]. *Gprc5a*-knockout (*Gprc5a*^−/−^) mice develop spontaneous lung tumors and are more susceptible than wild-type mice to development of eosinophilic macrophage pneumonia [16]. Moreover, *Gprc5a*^−/−^ mice were sensitive to inflammation-promoted lung tumorigenesis and endotoxin-induced acute lung injury [17, 18]. Thus, the causal relationship between silica exposure and neoplasia may be reproduced in *Gprc5a*^−/−^ mouse model that is susceptible to both pulmonary inflammation and lung tumorigenesis.

In this study, we examined and compared the biological response of lungs from both wild-type and *Gprc5a*^−/−^ mice following exposure to fine silica particles. The results show that the lungs of *Gprc5a*^−/−^ mice were not only susceptible to silica-induced inflammation, but also experienced tissue damage, fibrogenic response, and increased hyperplasia or neoplasia.

## RESULTS

### Lungs from *Gprc5a*^−/−^ mice are susceptible to silica-induced edema, lesions and inflammation

**Figure 1:**
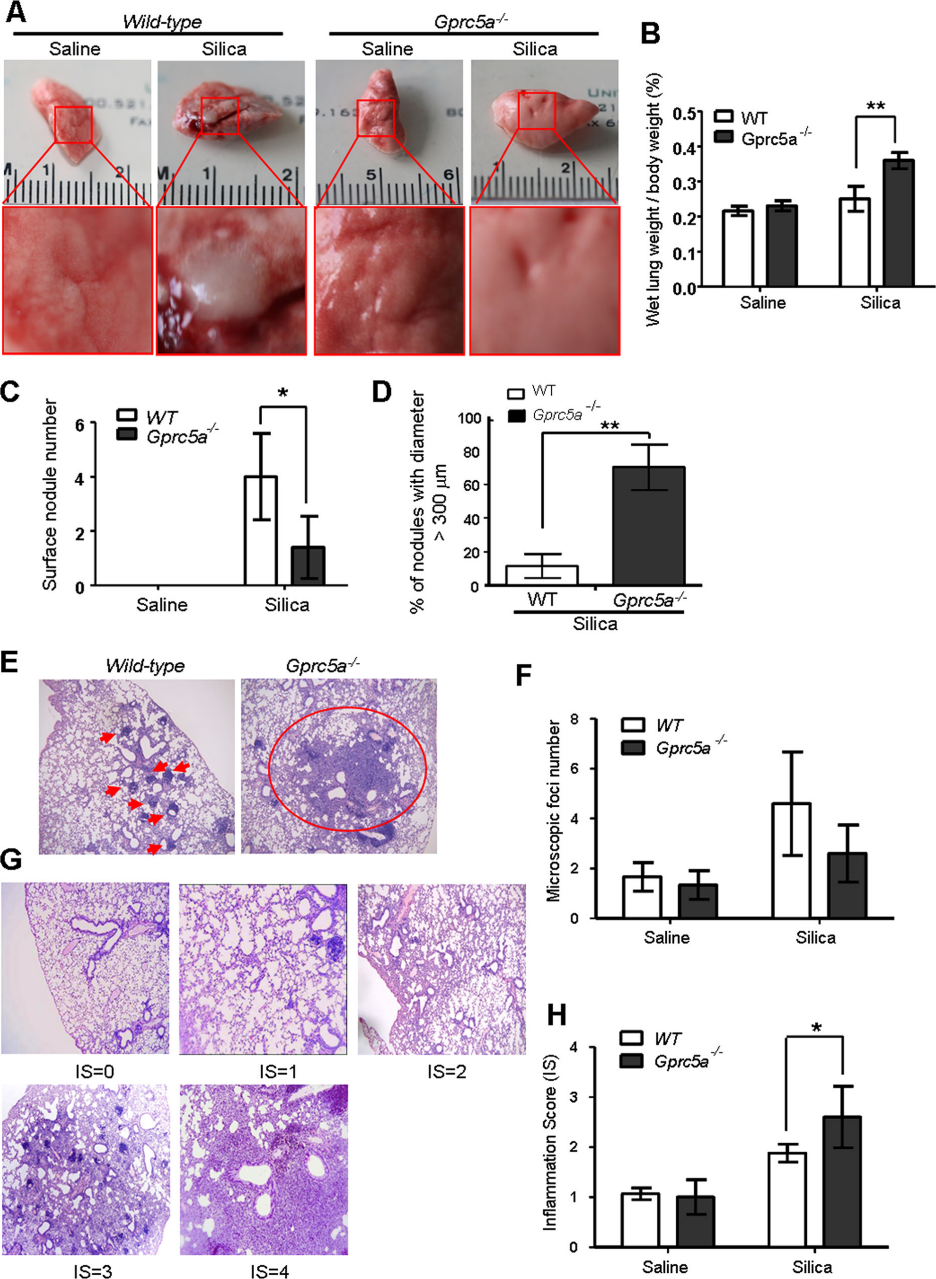
Lungs from *Gprc5a*^−/−^ mice are susceptible to silica-induced edema, injury and inflammation **A.** Representative images of lungs from wild-type and *Gprc5a*^−/−^ mice following treatment with silica (*n* = 5) or saline (*n* = 3) for 3 months. **B.** Bar graph indicates the ratio of wet lung weight over to body weight in tissues from wild-type and *Gprc5a*^−/−^ mice. **C.** Graph represents the number of surface nodules on lungs obtained from wild-type and *Gprc5a*^−/−^ mice with treatment as described above. **D.** Graph represents the percentage of nodules with diameter over 300 μm in lungs of wild-type and *Gprc5a*^−/−^ mice. **E.** Representative H&E images of lung tissues from wild-type and *Gprc5a*^−/−^ mice treated with saline or silica for 3 months. **F.** Graph represents the number of microscopic focal lesions on the lungs from wild-type and *Gprc5a*^−/−^ mice with treatments as indicated. Each lung section was systematically scanned at 100X magnification; five successive fields were counted. After examination of the entire section, the mean foci number from all examined fields was calculated and expressed as mean ± SEM. **G.** Representative H&E images of lung tissues with inflammation score (IS) from 0 to 4 in wild-type and *Gprc5a*^−/−^ mice following silica treatment. **H.** Graph of IS (F) was used as semi-quantitative assessment for pulmonary injury. *p < 0.05, **p < 0.01.

To investigate the biological effects of silica exposure, we first examined histological changes in lungs from wild-type (WT) and *Gprc5a*^−/−^ mice three months after intra-tracheal instillation of crystalline silica particles. Lungs from wild-type mice exhibited many but small nodules on the surface (Figure 1A). On contrast, lungs from *Gprc5a*^−/−^ mice showed large area of severe edema (Figure 1A) and increased wet lung weight (Figure 1B). Although there were more nodules in lungs from wild-type mice than in those from *Gprc5a*^−/−^ ones, the size (diameter) of nodules in lungs from *Gprc5a*^−/−^ mice were much bigger than those from wild-type ones (Figure 1C–1D). Consistently, H&E microscopic analysis showed many small and confined silica-induced lesions in the lungs of wild-type mice (Figure 1E–1F). In comparison, the lesions from the lungs of the *Gprc5a*^−/−^ mice were more spread and intense, and therefore graded with an increased inflammation score (IS) when compared to wild-type lungs (Figure 1G–1H). Taken together, these observations suggest that lung tissues from *Gprc5a*^−/−^ mice have an increased susceptibility to silica-induced lesions and inflammation, and have lost the capacity to confine the silica-induced lesions.

### *Gprc5a* deficiency exacerbates the silica-induced tissue damages and fibrogenic response in mouse lungs

**Figure 2:**
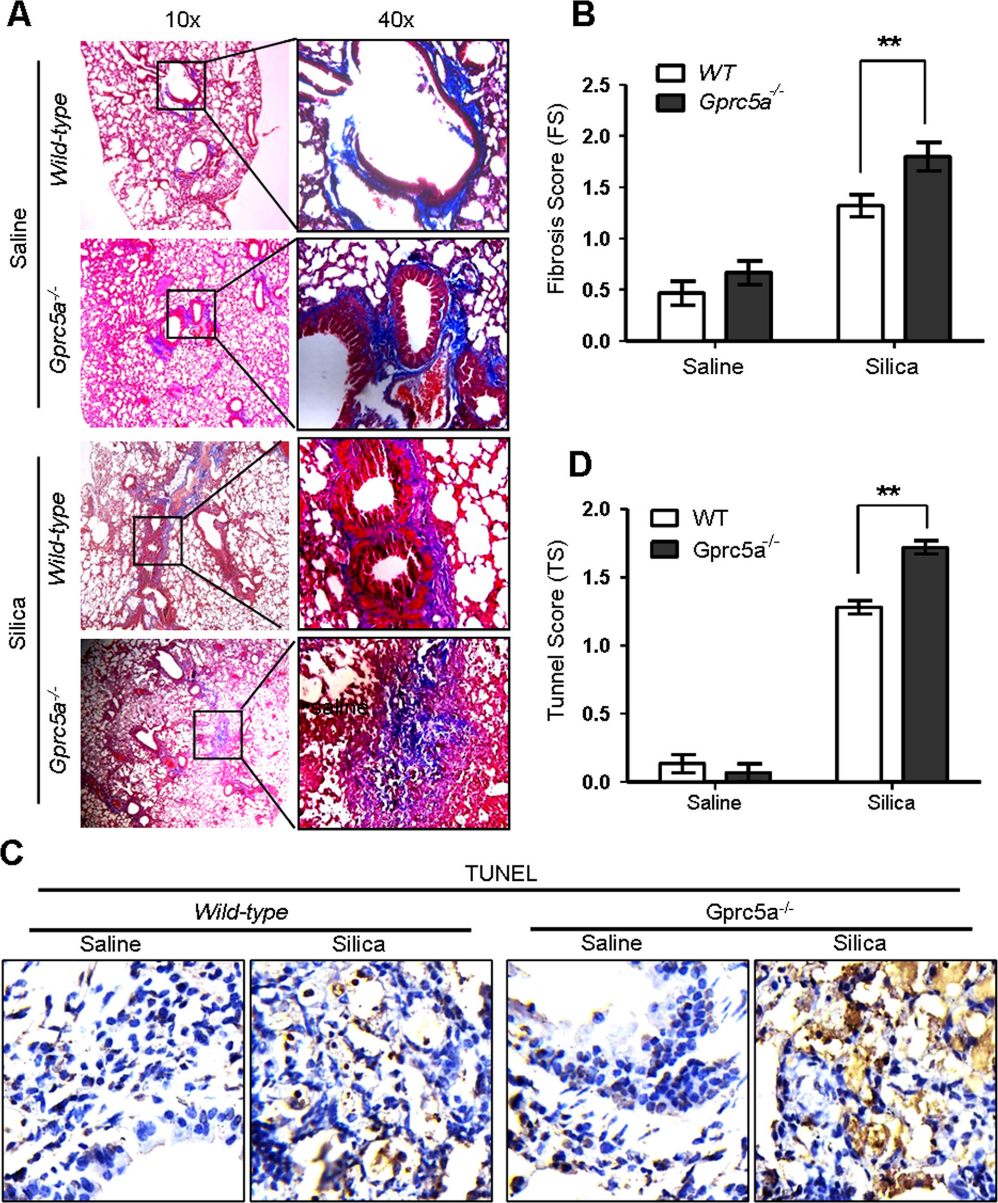
*Gprc5a* deficiency exacerbates the silica-induced tissue damages and fibrogenic response in mouse lungs **A.** Representative images of Masson staining, measuring collagen deposition, in lung tissues from wild-type and *Gprc5a*^−/−^ mice following silica (*n* = 5) or saline (*n* = 3) treatment for 3 months. **B.** Fibrosis score (FS) was used as semi-quantitative assessment for pulmonary fibrosis. The scoring method, outlined in the Methods section, is analogous to that used for inflammation (0–4) but based on Masson staining. *p < 0.05, **p < 0.01. **C.** Representative images of TUNEL staining in the lungs from *Gprc5a^+/+^* and *Gprc5a*^−/−^ mice following treatment schedule described above. **D.** Tunnel score (TS) was used as semi-quantitative assessment for apoptotic index. The scoring method, outlined in the Methods section is analogous to that used for inflammation (0–4). *p < 0.05, **p < 0.01.

Pulmonary fibrosis is a hallmark of inappropriate repair in response to chronic inflammation. The response is characterized by excessive extracellular matrix production, notably collagen deposition, and tissue destruction. Pulmonary fibrosis, or scar formation, appears as thickened alveolar walls, which causes reduced oxygen to blood. The silica-induced fibrogenic response produces nodular foci of collagen deposition, and is easily demonstrated by Masson staining. We found limited collagen deposition in untreated (saline) lungs from wild-type and *Gprc5a*^−/−^ mice, and that the collagen was well organized around bronchi and blood vessels, with minimal reactivity in the alveolar septi. In contrast, collagen deposition was increased dramatically in the lungs of silica-treated mice. Of note, lungs from silica-treated *Gprc5a*^−/−^ mice showed increased fibrosis scores, dispersed pattern of collagen deposition, destruction in bronchiolar and alveolar structure, compared to those from wild-type mice (Figure 2A–2B). Moreover, TUNEL analysis showed an increased apoptotic index in lungs from silica-treated *Gprc5a*^−/−^ mice compared to those from wild-type mice (Figure 2C–2D). Thus, an exacerbated tissue damage and fibrogenic response was elicited in lungs from *Gprc5a*^−/−^ mice with silica exposure.

### Silica exposure induces EMT characteristics in lungs from *Gprc5a*^−/−^ mice *in vivo*

**Figure 3:**
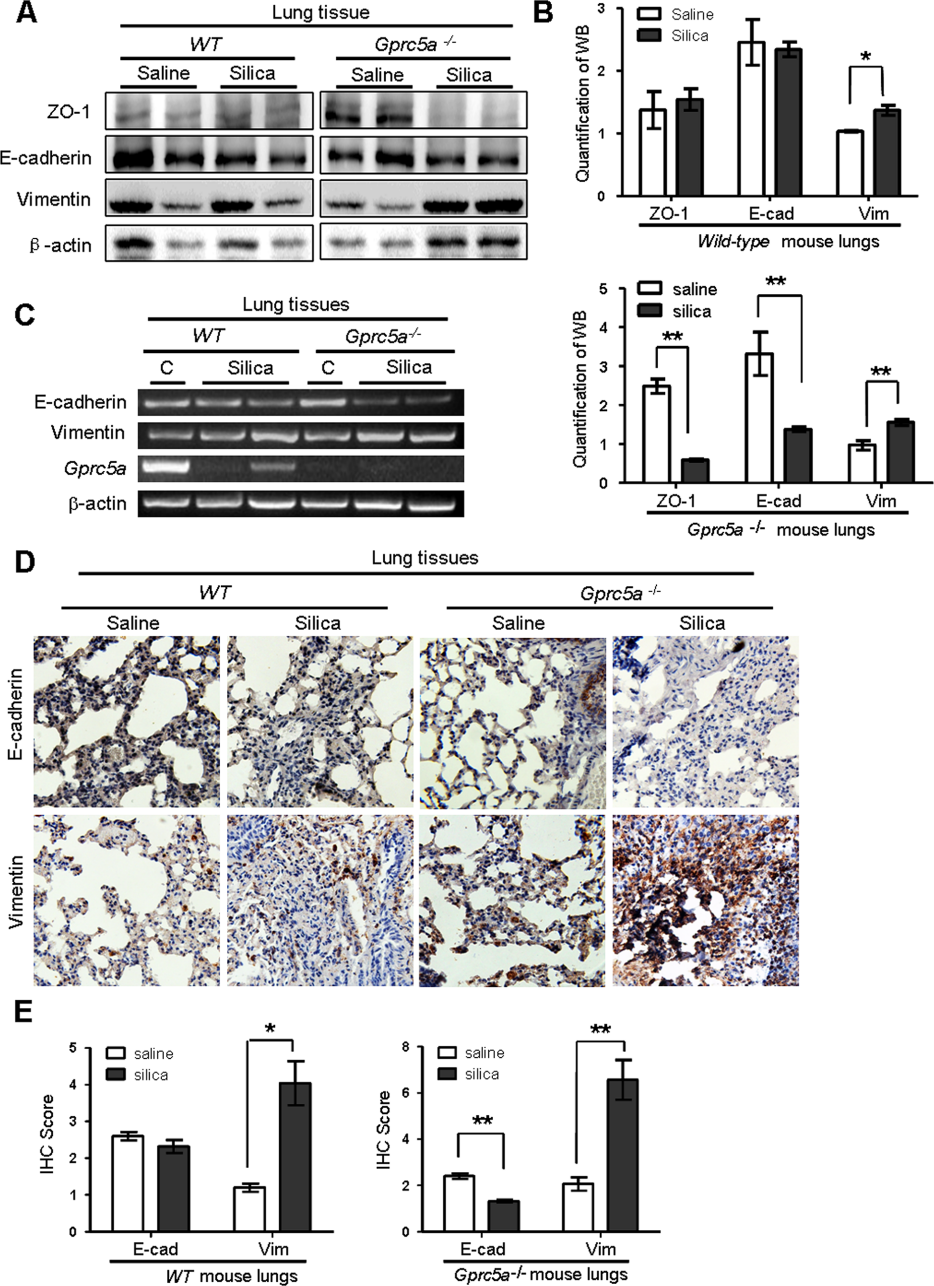
Silica exposure induces EMT characteristics in lungs from *Gprc5a*^−/−^ mice *in vivo* **A.** Western blot analysis for ZO-1, E-cadherin, and vimentin in lung tissue lysates obtained from wild-type and *Gprc5a*^−/−^ mice following treatment with silica or saline for 3 months. **B.** Graphic representation of estimated proteins levels obtained from Western blot in (A). **C.** RT-PCR analysis of mRNA expression (left) and quantified mRNA level (right) for E-cadherin and vimentin lung tissues obtained from wild-type and *Gprc5a*^−/−^ mice following treatment with silica or saline for 3 months. **D.** Representative images of IHC staining of E-cadherin and vimentin in lung tissue from wild-type and *Gprc5a*^−/−^ mice following treatment with silica or saline for 3 months. **E.** Bar graph represents the extent of IHC staining (IS) in (D). The scoring method (0–4) was described in the Methods section.

Lung epithelial cells are the targets of injury, and injury drives epithelial repair. An emerging concept is that epithelial-mesenchymal transition (EMT) plays a key role in the pathologic process of fibrotic lung diseases [19]. To determine if EMT characteristics are induced *in vivo*, we examined EMT-related markers in mouse lungs. Western blot analysis showed a significant decrease in expression for the epithelial tight-junction ZO-1 and cell adhesion markers E-cadherin in lungs from silica-treated *Gprc5a*^−/−^ mice, while the levels of vimentin, a mesenchymal cell marker, were increased significantly following silica treatment. Expression level changes for these proteins were relatively minor in lungs from silica-treated wild-type mice (Figure 3A–3B). Consistently, RT-PCR analysis showed that mRNA levels of E-cadherin was significantly suppressed, whereas vimentin was slightly increased in lungs from *Gprc5a*^−/−^ mice following silica treatment (Figure 3C). In comparison with, these markers remained relatively constant in lungs from wild-type mice (Figure 3C). The results were further confirmed by immunohistochemistry (IHC) staining, in which E-cadherin was suppressed whereas vimentin was increased in lungs from *Gprc5a*^−/−^ mice following silica exposure. In contrast, E-cadherin was not significantly suppressed in the lungs from wild-type mice, although vimentin was increased following silica exposure (Figure 3D–3E). Taken together, these results indicate that silica exposure induces EMT characters in lungs from *Gprc5a*^−/−^ mice *in vivo*, which is consistent with an exacerbated lung injury and fibrogenic response.

### Silica induces EMT-like characteristics in *Gprc5a*^−/−^ mouse tracheal epithelial cells (MTEC) *in vitro*

**Figure 4:**
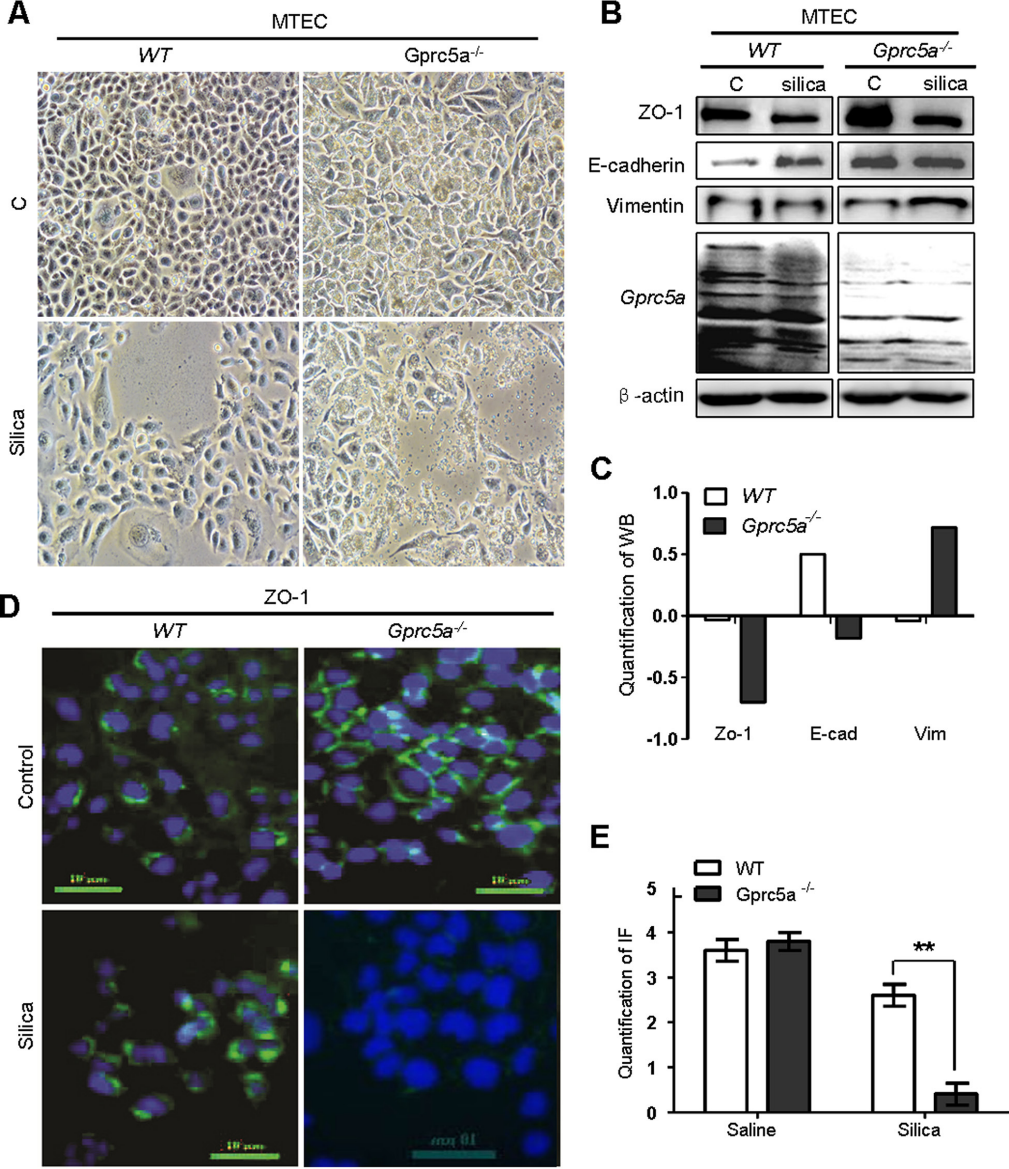
Silica induces EMT-like characteristics in *Gprc5a*^−/−^ mouse tracheal epithelial cells (MTEC) *in vitro* **A.** Representative images of cell morphology of *Gprc5a^+/+^* and *Gprc5a*^−/−^ MTEC treated without or with silica (500 μg/ml) for 72 h. **B.** Western blot analysis for ZO-1, E-cadherin, and vimentin in wild-type and *Gprc5a*^−/−^ MTECs treated with or without silica as indicated. **C.** Graphic representation of estimated proteins levels from Western blot (B). **D.** Representative images of immunofluorescence (IF) for ZO-1 in wild-type and *Gprc5a*^−/−^ MTEC and treated without or with silica (500 μg/ml) for 72 h. **E.** Graphic representation of estimated IF in (D).

Next, we examined the net effects of silica particles on mouse lung epithelial cells *in vitro*. Treatment of *Gprc5a*^−/−^ mouse tracheal epithelial cells (MTEC) with silica particles for 72 hours resulted in morphologic changes from epithelial to mesenchymal phenotype, as some cells became spindle shape, a fibroblast-like phenotype, while wild-type (WT) MTEC did not (Figure 4A). Moreover, Westernblots showed that, following silica-treatment, *Gprc5a*^−/−^ MTEC displayed increased expression of the mesenchymal marker vimentin, and significantly suppressed epithelial marker ZO-1 although E-cadherin was only slightly reduced (Figure 4B–4C). On contrast, wild-type MTEC only showed minor changes with the same treatment (Figure 4B–4C). These observations were confirmed by immunofluorescence (IF) analysis, in which ZO-1 was significantly suppressed in *Gprc5a*^−/−^ MTEC but not wild-type MTEC following silica exposure (Figure 4D–4E). Thus, *Gprc5a*-deficiency renders lung epithelial cells susceptible to silica-induced EMT characteristics, which is consistent with increased fibrogenic response *in vivo*.

### Recruitment of alveolar-macrophages was increased in lungs from *Gprc5a*^−/−^ mice

**Figure 5:**
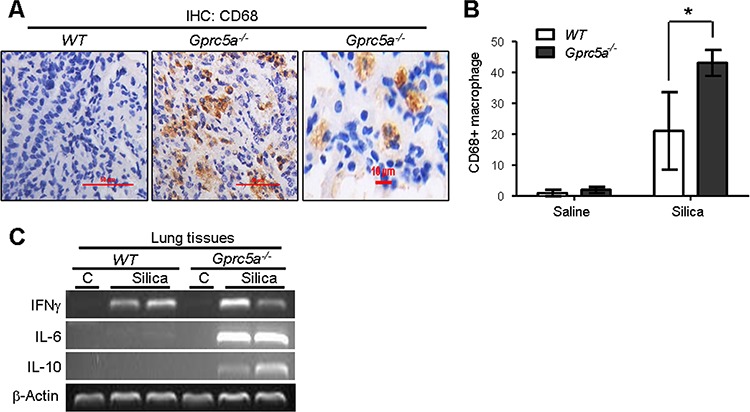
Recruitment of alveolar-macrophages was increased in lungs from *Gprc5a*^−/−^ mice **A.** Representative images of IHC staining for macrophage marker CD68 in lung tissue from wild-type and *Gprc5a*^−/−^ mice three months after silica exposure. **B.** Graph represents the extent of CD68 staining (IS) estimated from the IHC (A). The scoring method, outlined in the Methods section, used the 0–4 design. **C.** RT-PCR analysis of mRNA levels in lung tissues from wild-type and *Gprc5a*^−/−^ mice following silica exposure as indicated.

Recruitment of alveolar macrophages is involved in promotion of pulmonary inflammation in many circumstances. To determine if infiltrated alveolar macrophages were involved in silica-induced pulmonary inflammation observed in the lungs of *Gprc5a*^−/−^ mice, we examined expression of CD68, a macrophage marker, by IHC staining. We found a significant increase CD68 staining in lungs from *Gprc5a*^−/−^ mice compared to lungs from wild-type mice (Figure 5A–5B). This suggests that recruitment of alveolar macrophages is involved in pulmonary inflammation in *Gprc5a*^−/−^ mice following silica exposure. Two types of alveolar macrophages are involved in pulmonary inflammation. M1 macrophages produce the pro-inflammatory factors IL-12, IL-23, and TNFα, whereas M2 macrophages express IL-1, IL-6, and IL-10. RT-PCR analysis showed that M1 marker IFN-γ was induced in the silica-exposed lungs from both wild-type and *Gprc5a*^−/−^ mice (Figure 5C), whereas M2 markers, IL-6 and IL-10, were only dramatically induced in lungs from *Gprc5a*^−/−^ mice (Figure 5C). These results suggest that, while M1 macrophages were involved in pulmonary inflammation in both types of mouse lungs, M2 macrophages are more actively involved in the distinctive pulmonary inflammation in *Gprc5a*^−/−^ mice than in wild-type ones.

### Increased production of chemokines and proinflammatory cytokines in *Gprc5a*^−/−^ MTEC following silica exposure

**Figure 6:**
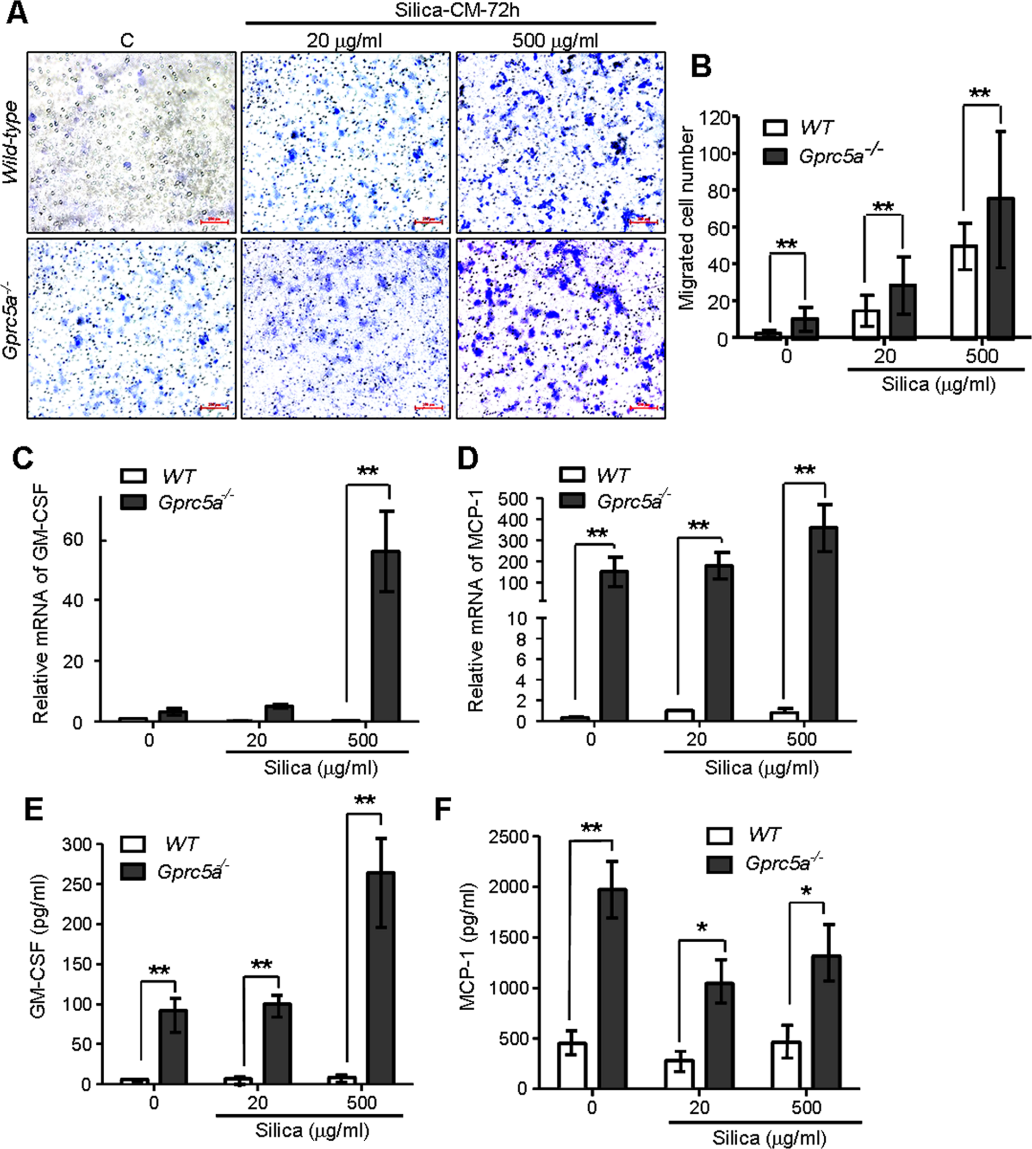
Increased production of chemokines and proinflammatory cytokines in *Gprc5a*^−/−^ MTEC following silica exposure **A.** Representative images of migrated MH-S cells on transwell membranes following co-culture with conditioned media from wild-type and *Gprc5a*^−/−^ MTEC that were pre-treated with different doses of silica for 72 h. **B.** Graph represents the number of MH-S cells migrated through transwell membrane. Presented data are the average ± SEM of cell counts from five successive fields at 100 × magnification. **C, D.** Q-PCR analysis of GM-CSF (C) and MCP-1 (D) expression in wild-type and *Gprc5a*^−/−^ MTEC following silica treatment as indicated. **E, F.** Protein chip analysis of GM-CSF (E) and MCP-1 (F) expression in cell-free supernatants of wild-type and *Gprc5a*^−/−^ MTEC pre-treated with different doses of silica over 72 h. *p < 0.05, **p < 0.01.

Chemokines produced by lung epithelial cells are the key factors for recruitment of alveolar macrophages. To investigate the causal factors for increased recruitment of alveolar macrophages in *Gprc5a*^−/−^ mice, we examined the conditioned medium from MTEC for induction of macrophage migration via transwell experiment. Conditioned media from silica-treated MTEC induced more macrophage migration than media from untreated cells; and the conditioned media from *Gprc5a*^−/−^ MTEC induced more macrophage migration than those from wild-type MTEC with the same treatment (Figure 6A–6B). Consistently, expression of GM-GSF (granulocyte-macrophage colony stimulating factor) and chemokine MCP-1 (monocyte chemotactic protein 1) was significantly increased in *Gprc5a*^−/−^ MTEC compared to that of wild-type by both Q-PCR and protein-chip analysis (Figure 6C–6F). Moreover, silica-exposure further increased the production of GM-CSF and MCP-1 (Figure 6C–6F). Taken together, silica exposure induced production of chemokines in MTEC; which likely contributes to increased recruitment of macrophage and pulmonary inflammation; whereas *Gprc5a* deficiency further increased the intensity of the response.

### Epithelial neoplasia coincides with exacerbated tissue damage and fibrogenic response in silica-exposed lungs from *Gprc5a*^−/−^ mice

**Figure 7:**
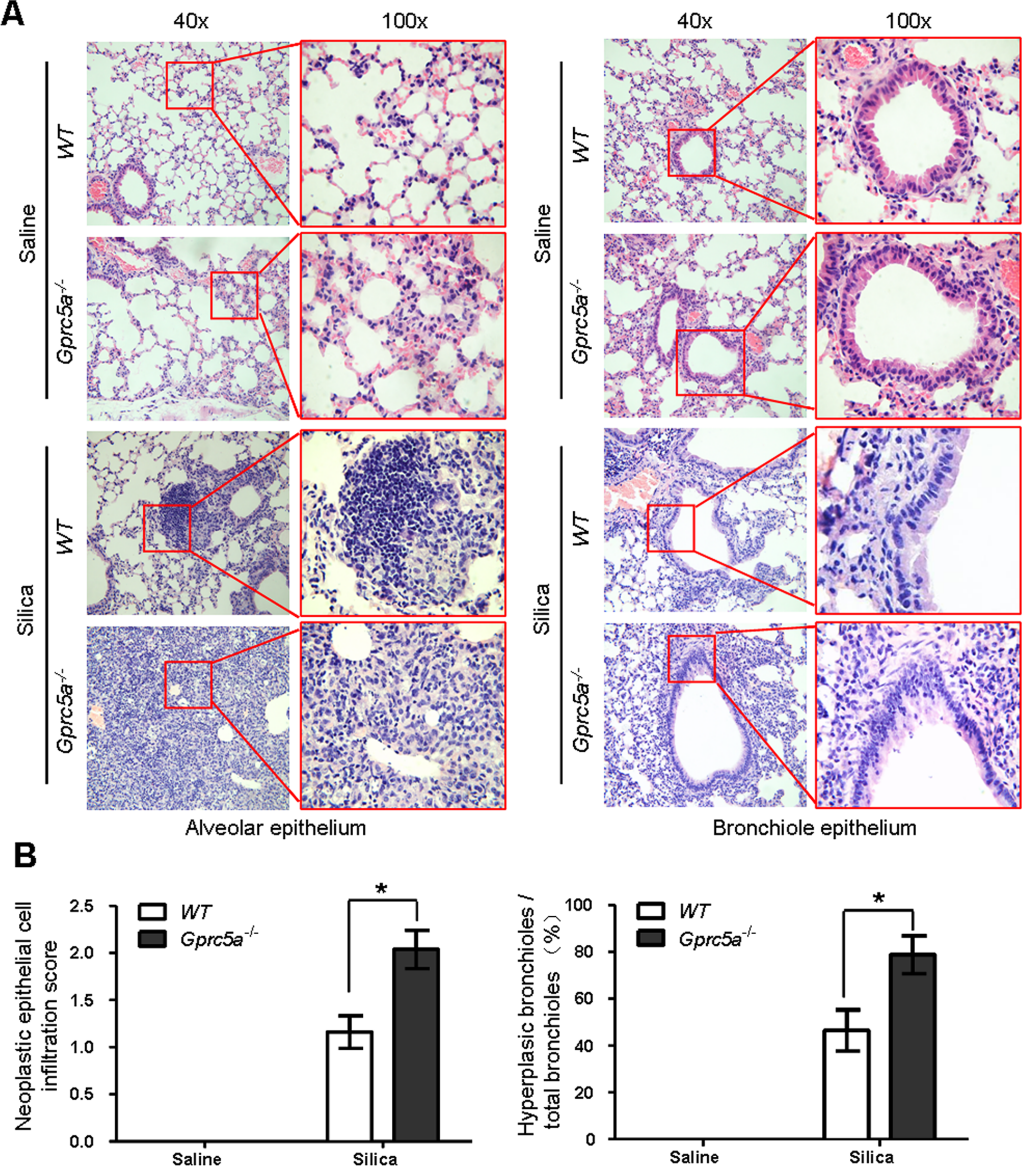
Epithelial neoplasia coincides with exacerbated tissue damage and fibrogenic response in silica-exposed lungs from *Gprc5a*^−/−^ mice **A.** Representative H&E images demonstrating the morphology of alveolar epithelium (left) and bronchi (right) obtained from wild-type and *Gprc5a*^−/−^ mice following silica treatment as indicated. The areas of neoplastic epithelial cell infiltration associated with immunoblasts and fibroblasts in silica-treated groups are shown in the red box (left). The bronchiolar epithelium hyperplasia in silica-treated groups is shown in the red box (right). **B.** Graphic representation of the extent of hyperplasia and abnormal bronchiole area in indicated treatments. The scoring method, outlined in the Methods section, used the 0–4 design.

H&E staining showed that there were significantly more lesions of neoplastic epithelial cell infiltration and bronchiolar epithelium hyperplasia of lungs from *Gprc5a*^−/−^ mice following silica exposure than those from wild-type or untreated groups (Figure 7A–7B). The areas of neoplastic epithelial cells infiltration in lungs from *Gprc5a*^−/−^ mice were exaggerated and more wide-spread than in wild-type lungs, which proportionally correlates with the intensity of lung tissue damage and fibrogenic response. Taken together, compared to those from wild-type mice, lungs from *Gprc5a*^−/−^ mice exhibited an increased neoplasia that coincided with increased tissue damages and fibrogenic response following silica exposure.

### Molecular program for neoplasia coincided with those for exacerbated tissue damage and fibrogenic response in lungs from *Gprc5a*^−/−^ mice following silica exposure

**Figure 8:**
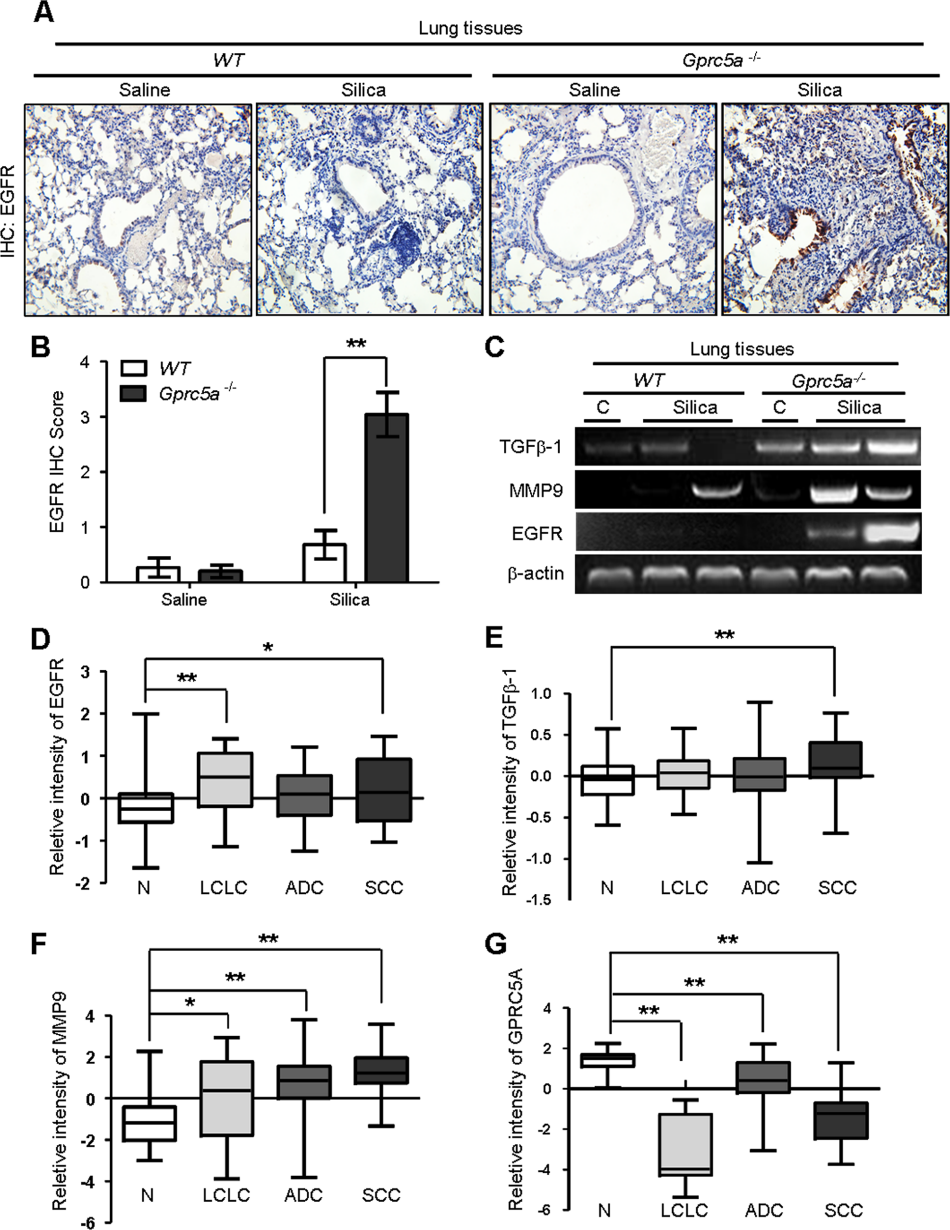
Molecular program for neoplasia coincided with those for exacerbated tissue damage and fibrogenic response in lungs from *Gprc5a*^−/−^ mice following silica exposure **A.** Representative images of IHC staining for EGFR in lung tissues from wild-type and *Gprc5a*^−/−^ mice, with or without silica treatment as indicated. **B.** Graphic representation of the extent of EGFR staining (IS) in (A) The scoring method, outline in the Methods section used the 0–4 design. **C.** RT-PCR analysis of mRNA expression for *TGFβ1*, *MMP9* and *EGFR* in lung tissue obtained from wild-type and *Gprc5a*^−/−^ mice following silica or saline treatment for 3 months. Relative mRNA levels for *EGFR* **D.** *TGFβ1* **E.** *MMP9* **F.** and *GPRC5A* **G.** in four different histological types of NSCLC: N: adjacent normal lung samples; LCLC: large cell lung cancer; LA: lung adenocarcinoma; SCC: squamous cell cancer. Data calculations are outlined in the Methods section. *p < 0.05, **p < 0.01.

EGFR promotes epithelial cell proliferation whereas up-regulated EGFR is linked to hyperplasia or neoplasia in lung epithelium. To correlate the biologic changes, we examined gene expression pattern related to those biological changes. IHC staining showed that silica exposure significantly increased EGFR expression in lungs from *Gprc5a*^−/−^ mice compared to those from untreated *Gprc5a*^−/−^ or wild-type mice (Figure 8A–8B). This suggests that lung epithelium from silica treated *Gprc5a*^−/−^ mice are more active in proliferation than other groups.

*MMP*s and *TGFβ-1* expression are linked to tissue injury, and fibrogenic response [20, 21], whereas EGFR is actively involved in tissue repair. Next, we examined expression of these genes by RT-PCR analysis. The results showed that, mRNA expression of *EGFR*, *TGFβ1* and *MMP9* were all significantly up-regulated in lungs from *Gprc5a*^−/−^ mice following silica exposure, whereas little change was found in lungs from untreated *Gprc5a*^−/−^, or either treated or control wild-type mice (Figure 8C). Thus, the gene expression pattern correlates with the pathological processes observed *in vivo*. Taken together, the gene expression program for neoplasia in the lungs of *Gprc5a*^−/−^ mice following silica exposure coincided with exacerbated tissue injury and increased fibrogenic response.

To expand the relevance of the conclusion obtained from mouse model to human tissues, we searched data from Oncomine (www.oncomine.com). The report from Hou et al [22], showed that expression of *EGFR*, *MMP9* and *TGFβ-1* were all upregulated in human lung cancer samples, including LCL (large cell lung cancer), ADC (adenocarcinoma) and SCC (squamous cell carcinoma), compared to adjacent normal tissues (N) (Figure 8D–8F). In contrast, the mRNA expression of *GPRC5A* was significantly suppressed in the tissues of LCL, ADC, and SCC compared to adjacent normal lung tissue (N) (Figure 8G). These results suggest that the gene expression pattern of injury, fibrogenic response and neoplasia are consistent with lung tumor development in human. Taken together, these results strongly support the model that hyperplasia or neoplasia coincide with the processes of lung injury and tissue remodeling.

## DISCUSSION

In this study, we investigated the pathological effects in *Gprc5a*^−/−^ mouse model following silica exposure, and found that the risk of neoplasia was associated with exacerbated lung injury, increased pulmonary inflammation and severe fibrogenic response induced by silica exposure. This suggests that chronic respiratory exposure to silica or PM2.5 particles would increase the incidence of lung cancer development as well as lung tissue damages and fibrosis.

Previous studies failed to induce neoplasia and fibrosis in experimental model by silica-exposure, which was probably due to the insensitivity or low response of wild-type mice. Thus, application of *Gprc5a*^−/−^ mice in this study is important, which ensures that the pathological effects induced by silica exposure was greatly amplified or accelerated since *Gprc5a*^−/−^ mice are susceptible to lung tumorigenesis [15, 16, 23] and endotoxin-induced lung injury [18]. In another word, *Gprc5a* mouse model overcomes the limitation of experiment performed in wild-type mice.

Lung nodules are quite common in clinic. Usually, a large nodule is more likely to be cancerous than a smaller one [24]. Thus, small and confined nodules suggests that wild-type mouse lungs had the ability to confine or restrain the pathological effects of silica particles, whereas *Gprc5a*^−/−^ mouse lungs failed to do so, leading to formation of neoplasia. For induction of lung injury and fibrosis, although extrinsic factors, such as duration, total exposure, and content of free crystalline silica, are critical [2], intrinsic or genetic factors also affect the susceptibility of lung tissue to injury and fibrogenic response. There are many candidate genes implicated in pulmonary fibrosis, such as *IGF-I*, *IGFBPs* [25], *ELMOD2*, *TERT*, *TERC*, *SFTPC*, SFTPA2), and MUC5B [26]. And specific HLA haplotypes were implicated to associate with the susceptibility for development of pulmonary fibrosis [7]. Also, inbred strains of mice differ in their susceptibility to fibrogenic agents [2]. Here, we showed that *GPRC5A* genotype is a factor affecting host susceptibility to silicosis.

There are more than 40 types of cells in lung tissues. Epithelial cells interact with other resident cells in the lung, including innate immune cells, endothelial cells, and mesenchymal cells such as fibroblasts [19] [27]. Lung epithelial cells serve as a functional unit for gas exchange and a physical barrier to protect cells from damaging environmental substances. When silica particles, or other inhaled fine particles, deposit directly into the lower respiratory airways, alveolar epithelial cells are injured, which triggers the secretion of proinflammatory cytokines and chemokines [28]. Previously, we showed that *Gprc5a*^−/−^ MTEC produced more chemokines than wild-type ones, including MCP-1, RANTES, MEC, M-CSF, CROα, CROβ, ENA78, and IP-10, which was attribute to aberrantly activated NF-κB in *Gprc5a*^−/−^ MTEC [29]. Increased production of chemokines is likely responsible for increased infiltration of macrophages in *Gprc5a*^−/−^ mouse lungs following silica exposure. Macrophages, especially apoptotic macrophages, have been reported to induce pulmonary inflammation and fibrosis [30, 31]. Moreover, recruitment of M2 macrophages has been linked to promotion of lung cancer development [32]. Thus, increased M2 macrophages in lungs of silica-exposed *Gprc5a*^−/−^ mice is consistent with increased tissue damages and fibrogenic response in these tissues. Previously, in the model of endotoxin-induced acute lung injury (ALI), NF-κB target gene expression was greatly activated in lungs from *Gprc5a*^−/−^ mice compared to wild-type ones following administration of endotoxin, with a peak of activation within 2–24 hour [18]. However in this study, the mouse model is silica-induced lung injury, in which the samples of lung tissues were collected three months after silica exposure. We also examined the expression of several NF-κB downstream target genes such as CCND, VEGF, and IκBα via Q-PCR analysis, but no difference was found in lungs from wild-type and *Gprc5a*^−/−^ mice before and after silica exposure (data not shown). This suggests that the chronic pathological effects on NF-κB pathway in lung by silica exposure are different from those of acute lung injury induced by endotoxin.

*Gprc5a*^−/−^ mice are more sensitive than wild type mice to silica-induced fibrogenic response in lung, not only in morphology such as collagen deposition, but also in gene expression pattern, including repression of epithelial cell markers, ZO-1, and E-cadherin, induction of mesenchymal marker vimentin, and expression of TGFβ and MMP9. Persistent exposure of alveolar epithelial cells to TGFβ has been shown to induce EMT, which accounts for the appearance of myofibroblasts in idiopathic pulmonary fibrosis [21, 33]. Thus, increased TGFβ expression likely contributes to increased fibrogenic response following silica exposure in pulmonary epithelial cells in *Gprc5a*^−/−^ mice. The expression pattern of *EGFR*, *TGFβ1* and *MMP9* in lungs from *Gprc5a*^−/−^ mice suggests that these programs concurred *in vivo*. It appears that persistent injury (MMP9 up-regulation) and inflammation (M2 macrophage infiltration) leads to increased fibrogenic response (TGFβ up-regulation) and dysregulated tissue repair (EGFR up-regulation). Thus, persistently up-regulated EGFR signaling for tissue repair appeared to be hijacked for neoplasia program in the lungs of *Gprc5a*^−/−^ mice following silica exposure.

Lung carcinogenesis has been considered as a multi-stage process in which pulmonary epithelial cells are mutagenized by carcinogens [34]. However, other factors, such as microenvironment and aging, also have significant impact on cancer incidence, as suggested recently by the evolutionary model of cancer [35]. From the points of view, the pathological effects, such as chronic inflammation, persistent injury, fibrogenic response, and structural changes, are accelerated aging processes which promote neoplasia or tumorigenesis [36, 37]. In other words, silica exposure resulted in an accelerated aging microenvironment favoring carcinogenesis in lung tissue from *Gprc5a*^−/−^ mice, but this effect was very limited or restrained in wild-type mice.

In summary, *Gprc5a* deficiency exacerbated the pathologic processes induced by silica exposure, with increased lung injury, persistent pulmonary inflammation, induction of EMT-like characteristics and neoplasia. These observations suggest that respiratory exposure of silica particles promotes lung tumorigenesis as well as persistent lung injury, tissue damages and fibrogenic response. Thus, prevention of respiratory exposure to silica or PM2.5 particles and chronic pulmonary inflammation would be helpful in reducing the incidence of lung tumorigenesis.

## MATERIALS AND METHODS

### Experimental animals and silica administration

*Gprc5a*^−/−^ mice were generated in a mixed background of 129sv × C57BL/6 as described previously [15]. Genotyping of mouse progeny was performed as described previously [16]. This study was carried out in strict accordance with the recommendations from the Guide for the Care and Use of Laboratory Animals of the National Institutes of Health. The protocol was approved by Shanghai Jiao Tong University School of Medicine Animal Care and Use Committee (experimental animal use permission No: SYXK (Shanghai) 2008–0050). All surgery was performed under general anesthesia, and all efforts were made to minimize suffering.

Crystalline silica particles (d50: 2.2 μm) were purchased from Shanghai Huijing Sub-Nanoseale New Material Co., Ltd. The particles were heated to 200°C for 2 h immediately prior to administration in order to sterilize the silica and inactivate any trace endotoxin. Eight-week-old male wild-type and *Gprc5a*^−/−^ mice received either silica (2.5 mg suspended in 60 μl of saline; *n* = 5) or saline alone (60 μl; *n* = 3) through laryngoscope. Previous studies indicated that this dose induced chronic lung inflammation and fibrosis [38]. All instillations were performed with 4% chloral hydrate anesthesia. At the termination of the experiment (3 months after silica installation) animals were sacrificed and lung tissues collected; wet lung weights were obtained and tissues processed for a variety of analyses. The left lung lobe was fixed with 4% paraformaldehyde in phosphate-buffered saline (pH 7.2 ~ 7.4) for 24 h. The remaining lung tissues were homogenated in liquid nitrogen for protein or RNA extraction.

### Cell culture and culture condition

Mouse tracheal epithelial cells (MTEC) derived from normal tracheas of 3-week-old *Gprc5a*^−/−^ and wild-type mice (C57BL/6 × 129sv) were described previously [16, 39]. The epithelial cells were grown in keratinocyte serum–free medium (K-SFM, Invitrogen) supplemented with epidermal growth factor (EGF, 5 ng/mL) and bovine pituitary extract (50 μg/mL, Invitrogen). The cell lines were karyotyped by G banding at the Institutional Molecular Cytogenetics Facility (MD Anderson Cancer Center, Houston, TX) and found to be of mouse origin.

### Cell migration assay

The trans-well cell migration system consists of cell culture inserts with an 8.0 μm pore size in a 24-well plate (BD BioCoat #354578, San Jose, CA). MH-S (mouse alveolar macrophage-like cell line) cells were re-suspended with fetal bovine serum (FBS)-free DMEM, and seeded on the insert membrane (2 × 10^4^ cells). Conditioned media from wild-type or *Gprc5a*^−/−^ MTEC cells (5.4 × 10^5^), previously treated with different doses of silica (0, 20, 500 μg/ml) for 72 h were loaded in the lower chamber and the system maintained for 48 h. Migrated cells, which attached to the lower side of the filter, were fixed with 96% ethanol for 30 minutes and stained with 1.5% crystal violet. Migrated cells in five 100× microscopic fields were counted using a Nikon fluorescence microscope.

### Hematoxylin-Eosin (H&E), Masson and TUNEL Staining

Fixed lungs were embedded in paraffin, and sequential 5 μm sections were processed for H&E, Masson or TUNEL staining. Each section of lung samples was systematically scanned at 100 × magnification, 5 fields/slide, and graded according to the degree of inflammatory infiltration or the area of involved fibrosis. Grade 0 = normal tissue while grades 1~4 indicated the presence of pulmonary inflammation, collagen deposition (Masson stain) or TUNEL positive cells: 1 (< 25% of the slide), 2 (25% to 50% of the slide), 3 (50% to 75% of the slide), or 4 (>75% of the slide). After examination, the mean score from all examined fields was calculated and reported as the inflammation score (IS) or fibrosis score (FS) or TUNEL score (TS). Semi-quantifications of neoplastic epithelial cell infiltration were also graded according to the extent of pathology as described above.

### Analysis of mRNA expression by RT-PCR and Q-PCR

Total RNA from lung tissues was extracted with Tri-Pure Isolation Reagent (Roche, Switzerland) and cDNA prepared from 1 mg of total RNA using the SuperScript III System (Invitrogen Life Technologies). mRNA transcripts were identified by RT-PCR (Reverse Transcription-Polymerase Chain Reaction), using the following primers (mouse origin):

*Gprc5a* (F: 5′-GACACACTCTATGCACCTTAT TC-3′ and R: 5′-ACAGACCTTGTCTACTCCAG-3′); internal standard β-actin (F: 5′-AACAGTCCGCCTAG AAGCAC-3′ and R: 5′-CGTTGACATCCGTAAAGA CC-3′); E-cadherin (F: 5′-CCAAGCACGTATCAG GGTCA-3′ and R: 5′-ACTGCTGGTCAGGATCGTTG-3′); vimentin (F: 5′-CTGCGAGAGAAATTGCAGGAG-3′ and R: 5′-CTTTCATACTGCTGGCGCAC-3′); MMP9: (F: 5′-TGTCATCCAGTTTGGTGTCG-3′ and R: 5′-TGC CGTCCTTATCGTAGTCA-3′); TGF-β1(F: 5′-CTGCTGC TTTCTCCCTCAAC-3′ and R: 5′-GCGAGCCTTAGT TTGGACAG); IFN-γ (F: 5′-GGCAAAAGGATGGTG ACATGA and R: 5′- ACCTGTGGGTTGTTGACCTC); EGFR (F: 5′- CCACTTTGCCTTCACTCCTC and R: 5′- TCTCCAACAGATTGCCCAGT).

The Q-PCR (Quantitative Real-time PCR) reaction proceeded using the Applied Biosystems 7500 Fast Real-Time PCR System. Primers including Csf2 (GM-CSF) and Ccl2 (MCP-1) were purchased from GeneCopeia^™^. Mouse Actin was used as an internal control gene. The expression data was normalized to actin and quantified using the 7500 Fast System Software.

### Western blot analysis

Fresh tissues or cells were lysed with lysis buffer (20 mM Tris (pH 7.5), 1 mM EDTA, 150 mM NaCl, 1 mM EGTA, 1 mM β–glycerophosphate,1% Triton X-100, 2.5 mM sodium pyrophosphate, 1 mM Na_3_VO_4_, 4 μg/ml aprotinin, 4 μg/ml leupeptin, 4 μg/ml pepstatin, and 1 mM PMSF). Protein samples were separated by 10% SDS-PAGE and electrophoretically transferred to a nitrocellulose membrane. Nonspecific protein binding was blocked with 1 h incubation in 5% nonfat dried milk in Tris-buffer saline containing 0.1% Tween 20 (TBS-T) at room temperature (RT) with agitation. The nitrocellulose membrane was incubated with the primary antibodies: anti-GAPDH (Kangwei, China), anti-ZO-1 (Abcam), anti-vimentin and anti-E-cadherin (Santa Cruz Biotechnology) for overnight at 4°C rinsed with TBS/TBS-T, and subsequently incubated with IR Dye-conjugated secondary antibodies (Rockland Immunochemicals, Gilbertsville, PA) for 1 h at RT. Images were quantified using the Odyssey infrared imaging system (LI-COR Biosciences Lincoln, NE). The protein content was normalized to the level of GAPDH.

### Immunohistochemistry (IHC)

The samples of lung tissues were fixed with formalin buffer and embedded in paraffin. IHC staining was performed on 5 μm de-paraffinized-tissue sections with rabbit monoclonal antibody to: E-cadherin (Cell Signaling), vimentin (Cell Signaling) or EGFR (Cell Signaling) for overnight at 4°C. Tissue sections were incubated with biotinylated rabbit immunoglobulin G (for overnight at 4°C), followed by conjugated HRP streptavidin, (both) for 1 hr at RT, and DAB working solution until localization was visualized, with rinses between each incubation, and finally counterstained with hematoxylin. To quantify protein expression, the mean percentage of positive cells was determined in at least five random fields/slide at × 400 magnification and scored as follows: 1 (<25% of the slide), 2 (25% to 50% of the slide), 3 (50% to 75% of the slide), or 4 (>75% of the slide). The intensity of the detected immunoreaction was scored as follows: 1+, weak; 2+, moderate; and 3+, intense. The immunohistochemical staining score (IS) was calculated as the percentage of positive cells multiplied by staining intensity. These judgments were made by two independent pathologists.

### Immunofluorescence (IF)

Cells treated with silica (500 μg/ml) or PBS (control) for 72 h were fixed with cold methanol for 10 minutes and blocked with 5% FCS. The cells were incubated with primary antibodies (ZO-1 Abcam, 1 μg/ml) overnight at 4°C. Secondary antibodies were added to rinsed cells and incubated at 37°C for 1 h. FITC-conjugated rabbit IgG antibodies (Santa Cruz Biotechnology) identified positive staining and washed cells were stained with DAPI and viewed with a fluorescence microscope (Nikon).

### Assessment of chemokines

Wild-type and *Gprc5a*^−/−^ mouse tracheal epithelial cells (MTEC) (2 × 105 cells) were treated with various doses of silica (0, 20, 500 μg/ml) for 72 h. Cytokine levels in conditioned media from treated cells were measured using the Raybio^®^ Mouse Cytokine Antibody Array III according to the manufacturer's instructions (RayBiotech, CA. USA)

### Analysis of raw data in a public database

Expression levels for *GPRC5A*, *MMP9* and *EGFβ-1* were determined in 91 non-small cell lung carcinoma and 65 adjacent normal lung samples based on available raw data published as supporting information by Hou et al. [22]. We extracted the expression value of *GPRC5A*, *MMP9* and *EGFβ-1* from this dataset. The values used for gene expression were rescaled, and negative values were floored to 0, as described by the authors.

### Statistical analysis

Presented data are the mean ± SEM with statistical analysis performed using the SPSS,19.0 statistics package (SPSS Inc., Chicago, USA). Results were compared using unpaired *t*-tests, assuming unequal distribution, and statistical significance was set at *p* < 0.05.
